# Collaborative and partnership research for improvement of health and social services: researcher’s experiences from 20 projects

**DOI:** 10.1186/s12961-018-0322-0

**Published:** 2018-05-30

**Authors:** M. E. Nyström, J. Karltun, C. Keller, B. Andersson Gäre

**Affiliations:** 1 Department of Learning, Informatics, Management and Ethics, Medical Management Centre, Karolinska Institutet, SE 171 77 Stockholm, Sweden; 2 Department of Public Health and Clinical Medicine, Epidemiology and Global Health, Umeå University, SE 901 87 Umeå, Sweden; 3 Department of Industrial Engineering and Management, School of Engineering, Jönköping University, P.O. Box 1026, SE 551 11 Jönköping, Sweden; 4 Jönköping International Business School, Jönköping University, P.O. Box 1026, SE 551 11 Jönköping, Sweden; 5 Futurum, Region Jönköping County, Sweden; 6 The Jönköping Academy for Improvement of Health and Welfare, School of Health Sciences, Jönköping University, P.O. Box 1026, SE 55111 Jönköping, Sweden

**Keywords:** Collaborative research, co-production, integrated knowledge translation, partnership research, quality improvement, healthcare, social services

## Abstract

**Background:**

Getting research into policy and practice in healthcare is a recognised, world-wide concern. As an attempt to bridge the gap between research and practice, research funders are requesting more interdisciplinary and collaborative research, while actual experiences of such processes have been less studied. Accordingly, the purpose of this study was to gain more knowledge on the interdisciplinary, collaborative and partnership research process by investigating researchers’ experiences of and approaches to the process, based on their participation in an inventive national research programme. The programme aimed to boost collaborative and partnership research and build learning structures, while improving ways to lead, manage and develop practices in Swedish health and social services.

**Methods:**

Interviews conducted with project leaders and/or lead researchers and documentation from 20 projects were analysed using directed and conventional content analysis.

**Results:**

Collaborative approaches were achieved by design, e.g. action research, or by involving practitioners from several levels of the healthcare system in various parts of the research process. The use of dual roles as researcher/clinician or practitioner/PhD student or the use of education designed especially for practitioners or ‘student researchers’ were other approaches. The collaborative process constituted the area for the main lessons learned as well as the main problems. Difficulties concerned handling complexity and conflicts between different expectations and demands in the practitioner’s and researcher’s contexts, and dealing with human resource issues and group interactions when forming collaborative and interdisciplinary research teams. The handling of such challenges required time, resources, knowledge, interactive learning and skilled project management.

**Conclusions:**

Collaborative approaches are important in the study of complex phenomena. Results from this study show that allocated time, arenas for interactions and skills in project management and communication are needed during research collaboration to ensure support and build trust and understanding with involved practitioners at several levels in the healthcare system. For researchers, dealing with this complexity takes time and energy from the scientific process. For practitioners, this puts demands on understanding a research process and how it fits with on-going organisational agendas and activities and allocating time. Some of the identified factors may be overlooked by funders and involved stakeholders when designing, performing and evaluating interdisciplinary, collaborative and partnership research.

**Electronic supplementary material:**

The online version of this article (10.1186/s12961-018-0322-0) contains supplementary material, which is available to authorized users.

## Background

Healthcare organisations are complex and knowledge intensive, with patients often taking for granted that care providers use the best available knowledge on diagnosis and treatment. Evidence-based medicine and practice ensure that the best available knowledge is used systematically in clinical care (e.g. [1]). Nevertheless, the gap between research and practice in healthcare is well-known and a recognised concern (e.g. [2, 3]), where failures to translate research into practical actions contribute to health inequities [4, 5]. The process from research-produced knowledge to its use in healthcare practices can take considerable time (e.g. [6]). The estimated lack of research use in the United States and the Netherlands has suggested that 30–40% of patients do not receive care complying with current research evidence [7]. The required increase in the speed of uptake of evidence-based clinical practice guidelines has been frequently discussed (e.g. [8]) and factors influencing their adoption have been extensively studied (e.g. [9, 10]). Getting research into policy and practice in healthcare is a recognised, world-wide concern (e.g. [11]).

Several research areas deal with aspects related to the transfer of knowledge and use of research findings for improving healthcare. The view of research production as separate from the use of research findings initially inspired research on diffusion and implementation processes [12, 13], mainly focusing on the later stages of the research process with variable emphasis on a division between knowledge production and its implementation.

The shortcomings of the traditional ‘linear’ model of research-into-practice have become more evident [14]. Van de Ven and Johnson [15] suggest that the problem may be one of methods for knowledge production rather than knowledge transfer or knowledge translation. To enhance a faster and more systematic use of knowledge, collaborative and interdisciplinary research approaches have been asked for as well as more useful research [15–17]. Research collaboration is assumed to enable and enhance both the use of research and increase the amount of research relevant to end users.

Research on knowledge transfer and exchange describes an interactive exchange of knowledge between research users and researcher producers [18, 19]. Knowledge transfer and exchange interaction between researchers and practitioners can take place from the on-set of the research process and involve more long-standing relationships. Several approaches to knowledge transfer have been described, focusing, for example, on systematic synthesis and guidelines, social interaction between researchers and decision-makers, contextual features and organisational readiness [20]. The Canadian Institute of Health Research uses the term ‘integrated knowledge translation’ to describe projects where the knowledge users are involved as equal partners during the entire research process [21, 22].

Funding organisations have started to request research proposals to include researcher–decision-maker partnerships in collaborative research teams with representatives of industry, local communities and professional organisations (e.g. [23, 24]). The increased focus on research-use is also mirrored in research funders’ strategies (e.g. [25]). Some examples of collaborative initiatives are the Partnership projects and Centres financed by the National Health and Medical Research Council in Australia, the Dutch Academic Collaborative Centres for Public Health in the Netherlands, and The Collaborations for Leadership in Applied Health Research and Care in the United Kingdom (e.g. [26]), as well as the practice-based research networks and structured use of practice facilitators [27, 28] and the Integrated Delivery Systems Research Network programme to foster public–private collaboration between health service researchers and healthcare delivery systems in the United States [29]. There are three main strategies that research funding agencies might use to enhance knowledge translation – push, pull, or linkage and exchange. The push–pull strategies distinguish between mechanisms driven by science (push) and those driven by the demands of practitioners or policy-makers (pull) [30]. The linkage and exchange model is based on co-construction of applied knowledge and the relevance of applied research to both practitioners and researchers [31]. In a recent review of the ways research funding agencies support science integration into policy and practice in the field of health [30], most of the 13 agencies investigated used one or two of these strategies. The large heterogeneity of users and how this may affect the use of various mechanisms for research initiation, development and dissemination was highlighted in this review.

Collaboration has been addressed for some time in community-based participatory research regarding public health and social issues in society (e.g. [32, 33]), for example, on how to achieve policy level collaboration (e.g. [24, 34]) and evidence-informed policy-making (e.g. [35]). Nevertheless, there is a need for more empirical research on the actual processes, conditions and outcomes of the more recent collaborative and partnership research initiatives in healthcare [36, 37] and, to date, there are few empirical studies on researchers’ approaches and experiences of the combination of interdisciplinary and collaborative and partnership research, including the actual effect of such programme or project calls. Less explored is also research partnership aiming to respond to the challenges and priorities of the health system and much research has been based on assumptions of researcher-driven initiatives with newly established collaborations [38].

## Collaborative and partnership research

### Approaches, strategies and roles

Collaboration and partnerships are two concepts used to describe the involvement of people and groups from different contexts and with different experiences, perspectives and agendas in research and development. Accordingly, collaborative research contains social relations and a variation of potential roles for those involved during the research process. In earlier research, means for collaboration were described in the form of ‘linkage mechanisms’ between researcher and user contexts, i.e. the presence of intermediaries (boundary spanners); formal and informal contacts with users during studies; involvement of users during data collection; and interim feedback [39]. The boundary-spanning role of knowledge brokers has been brought forward as a bridge between research and practice (e.g. [40]). Knowledge brokering has been defined as “*all the activity that links decision-makers with researchers, facilitating their interaction so that they are able to better understand each other’s goals and professional cultures, influence each other’s work, forge new partnerships, and promote the use of research-based evidence in decision-making*” ([40], p. 131). Individuals, teams or organisations can all play the role of knowledge brokers [41, 42]. Michaels [43] describes six primary brokering strategies that span from more passive dissemination of information, interaction by seeking and using expert’s advice and linking different actors, to active engagements and close collaborative relations with healthcare actors, and which aim to inform, consult, match-make, engage, collaborate and build capacity. A recent study highlights the importance of effective ‘relationship brokering’ in researcher-health system partnership for establishing a meaningful collaboration [38].

A detailed road map on research collaboration is offered by Martin [44] in his description of five approaches to co-production of research. Depending on the chosen approach, stakeholders can be more or less involved in phases of the research process, from study design, data collection and analyses, to dissemination, while the degree of academic independence of the researcher/s and the utilisation of the research results may vary. Consequently, practitioners can play the role of informants, recipients, endorsers, commissioners or co-researchers.

On programme level, King et al. [45] describe four research programme operating models used in a collaborative approach to enhance research-informed practice in community-based clinical service organisations. The models describe the types of partnership involved such as the ‘clinician-researcher skills development model’, ‘clinician and researcher evaluation model’, ‘researcher-led evaluation model’, and the ‘knowledge-conduit model’. To differentiate research-practice partnerships from other ways of conducting research, Øvretveit et al. [46] suggest five criteria for partnership research, namely research that contributes to actions taken by actors within a health system; studies intended to produce quick and actionable findings as well as scientific publications; both researchers and practitioners take part in defining the research question and interpret findings; significant time and contributions from both researchers and practitioners; and an extensive formulated description of the partnership approach.

### Challenges and enabling features

One challenge for collaborative and partnership research concerns the variation of views on the production and use of knowledge and on the relationship between researcher and practitioner, spanning from top-down to bottom-up or from linear to interactive and multidimensional (e.g. [39, 47]). Depending on research tradition and/or experiences, basic assumptions regarding knowledge and learning can vary among researchers, but also among stakeholders (e.g. [48]). Sibbald et al. [49] identified challenges such as role clarity, organisational change and cultural differences regarding expectations on research output and (positive) effects on actual practice and found that role ambiguity, multiple roles and role conflicts could hamper social relationships. Factors facilitating collaboration were already established relationships, the alignments of goals/objectives, skilled and experienced researchers, and the use of regular, multi-modal communication.

Another influence on collaboration, mainly from the researcher context, is the variety of research paradigms and areas and related basic assumptions. One approach to be expected is action research, where knowledge creation is combined with practice development. There are a variety of action research approaches depending on, for example, how the collaborative element is organised [50]. The interactive research approach builds on action research and emphasises the common learning and knowledge creation for both practitioners and researchers during the complete process [51]. Both approaches involve a number of different roles for the researcher to enact [52].

Based on a realist evaluation, Rycroft-Malone et al. [36] list features of research collaboration likely to enhance knowledge use, namely attention to communication mechanisms, setting intermediate/outcome goals, providing time and space for the development and implementation of plans, making the choice of topic with resonance and relevance, close proximity between partners, re-balancing and sharing power, and allowing time to develop mutual trust and respect. These features put other demands on the planning and execution of research than a traditional approach when research has precedence over practice.

**Fig. 1:**
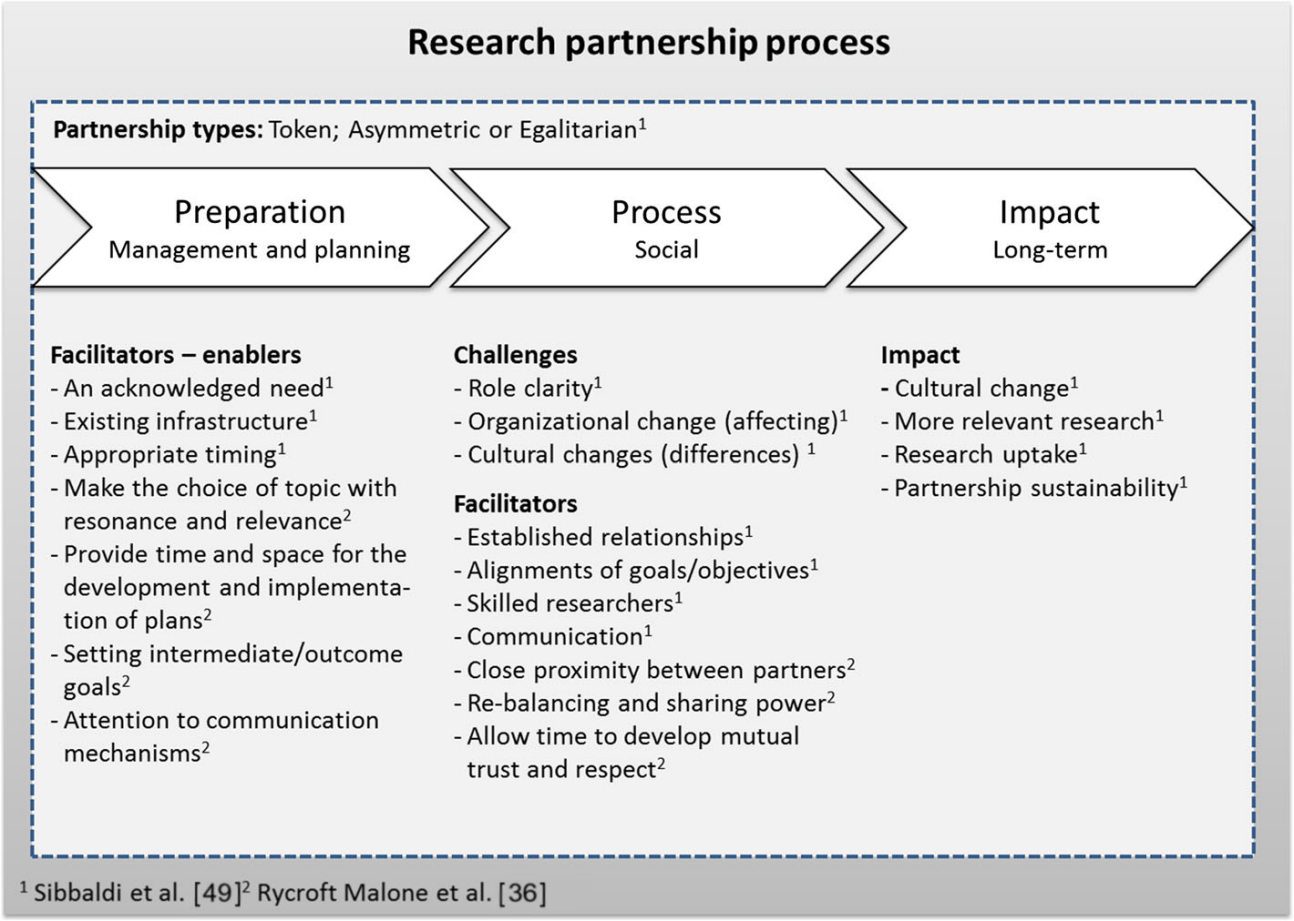
Model over the research partnership process (adopted after Sibbald et al. [49] and Rycroft Malone et al. [36])

Sibbald et al. [49] present a model describing the research partnership process, with enablers, facilitators, challenges and impact, and identify three partnership types based on an empirical study as token, asymmetric or egalitarian partnerships. In Fig. 1, features of the research partnership process are presented, grounded on aspects highlighted by Sibbald et al. [49] and Rycroft Malone et al. [36].

Collaborative and partnership research poses specific demands regarding project management. According to a review [53], collaborative research can be characterised by heterogeneity of actors, collective responsibilities, demands for applicability in addition to scientific requirements, and by being funded by public agencies with specific agendas. Project management in collaborative projects usually involves three paradoxes [53]:

1. To reach expected results, both freedom and flexibility to handle the uncertainty in research is needed, as well as a tight and managed firm project structure.
2. The necessary integration of different views of the actors involved may also lead to intercultural, interorganisational and interdisciplinary problems that need to be managed.
3. The limited formal authority of the project manager is in contrast with the demands for integrative managing of results, commitment and involvement of all parties.

Thus, collaborative and partnership research may have the potential to enhance the use of knowledge in practice and thereby improve healthcare and social services, but also to challenge the practitioners’ and researchers’ views, assumptions and roles.

The empirical base of the present study is an example of a collaborative and interdisciplinary research initiative – a national research programme seeking to boost collaborative and partnership research to improve health and social services in Sweden. The purpose of the study was to gain more knowledge on the interdisciplinary, collaborative and partnership research process by investigating researchers’ experiences based on their participation in this national programme. We have studied the experiences of researchers in all 20 research projects funded by the programme during the period 2008–2014, focusing on three themes – complexity in collaboration, collaborative procedures, and challenges, obstacles and lessons learned. The research questions posed were (1) what types of research approaches, research focus and partners/actors were involved in the projects? (2) How was collaborative and partnership research achieved, according to the researchers? (3) What were the challenges and obstacles to interdisciplinary, collaborative and partnership research encountered, and what main lessons were learned?

## Methods

This study is based on analyses of interviews and archival data. The interviews were performed during the final stages of the projects and the documents covered the entire project period.

### Empirical setting – the national research programme

The Vinnvård research programme was financed by a consortium of research funders, including the Ministry of Health and Social Welfare, Sweden’s innovation agency VINNOVA, Vårdalstiftelsen and the Swedish Association of Local Authorities and Regions. It was the first major attempt by research funders in Sweden to address the challenges of the ‘quality chasm’ in health and social services focusing on organisational aspects, with clear aspirations to bridge research and practice, stimulate interdisciplinary research and enhance research collaboration between universities/research institutions and healthcare/social service organisations. In addition, new research and learning infrastructures were expected to emerge. The requirements and evaluation criteria for the applications reflected these aspirations and documented active cooperation between practitioners, researchers and other public actors was asked for. Moreover, applications had to include an interdisciplinary approach, a common vision for all parties involved and documented approaches for securing participation and dissemination of research and/or development results. Both researchers and practitioners/public institutions could apply for funding. Non-researchers were part of the peer-review panel in both calls. The programme’s broad approach was new, both to the funding agencies and the research community. With a focus on health and social services, the programme aimed to (1) increase the use of research-based knowledge (bridge research and practice), (2) develop innovative ways of organising work, (3) stimulate the development of institutional learning structures with a focus on how to lead, manage and develop practices in organisations, and (4) establish more research on how to lead, manage and develop practices in health and social services organisations at Swedish universities.

The subsequent aims (3 and 4) had a more long-term character than the preceding ones (aims 1 and 2) and can be seen as a means to build long-lasting support for these. During the period studied (2008–2013) there were two calls for projects lasting up to 4 years each. A total of 20 projects were funded, 9 in 2008–2011 and 11 in 2009–2013, all of which are included in the present study. All projects were on-going when the interviews were conducted and all except one had ended when the archival data was gathered in May 2015.

### Data collection

Interviews were conducted between September and November 2011, when most projects were in their later stages. In total, 17 respondents with an equal gender distribution were chosen based on their overall project involvement. Four respondents were responsible for two projects each and one project was represented by two respondents. All 20 projects were covered. All respondents held a PhD and were intensely involved in the projects, either as project leaders, principal investigators, or as the most important researcher, as judged by the project leader. The semi-structured interviews had the aim of following up on the main goals and lessons learned within the projects and covered the characters of the projects with its overall contributions, roles taken in projects, collaborations, and participating or studied organisations and institutional levels. Questions followed five themes for each one of the four programme goals, namely interpretations of the goal, the importance it was given, how it was fulfilled, how the project had worked to reach the goal, and results related to the goal. Practical examples of the last two themes were requested. The last part of the interview covered how the four goals were integrated, any expected or unexpected insights, difficulties experienced, and emphasised and miscellaneous findings. In a few cases, interviews were complemented by the respondents with written comments or documents. Interviews lasted 40 to 120 min, with longer interviews with respondents representing two projects. The respondents received the questions a week in advance. Each interview was recorded and transcribed verbatim.

Archival data were gathered in May 2015 and consisted of the projects’ final reports and, for one project not yet finished, a progress report (same structure as the final reports, but without a popular science description and financial report). The report template had the following headings: introduction; short summary on how the project had worked to fulfil the research programme’s goals (with subheadings for each of the four goals); publication list; participation in national and international conferences/workshops; PhD students; description of potential problems encountered; description of the most important lessons learned; popular science description; references; and financial accounting. Reports varied in length from 4 to 42 pages, with an average of 19 pages; yet the four-page report came from the project not yet finished. A total of 386 pages were analysed.

### Data analysis

Qualitative data in interviews and documents were scrutinised in several steps, first by using a mix between directed (guided by the research questions) and conventional content analysis [54]. The entire material was read through to get a sense of the whole and then analysed to identify relevant text related to the research questions. Both data sources were then further used for determining themes, categories and overall patterns. Finally, a summative content analysis [54] was applied to get a sense of variation by identifying how many projects provided information in the identified categories. Three researchers performed the analyses, first individually and through meetings held to discuss and validate interpretations.

Document analysis focused on information related to the three research questions, namely (1) system level actors involved (care recipient, unit/clinic/ward, organisation, region/county council, and/or national level) and basic project information; (2) descriptions of or activities and strategies for research collaboration, including with which actors; and (3) obstacles/problems encountered and main lessons learnt. A fourth category (Other) was used in order not to miss important aspects. Described collaborations and process reflections (i.e. obstacles/problems and lessons learnt) were the main categories used to sort the material for questions 2 and 3. In a second iterative step for these questions, subcategories were identified, tested, revised and defined. The category definitions were then used in a third step for a final text classification of data.

As the interviews were performed when the projects were still ongoing, they were used to complement, add details, provide examples and validate information found in documents. Moreover, they also added a more personal perspective on the results based on the experience of the main researchers.

## Results

### Project overviews and the complexity of the collaborations

Of the 20 projects, 14 included three or more stakeholder levels, indicating an elaborated multilevel or system view. Five projects actively involved care recipients (and sometimes their next-of-kin) in design of solutions or interactive co-production. Two projects had a sole clinical focus (screening for atrial fibrillation and improving methods for stroke care), but such foci were present in several projects’ sub-studies. Four projects (three geographical sites) had a deliberate strategy to build learning structures that involved university level education at undergraduate and/or master’s levels, but education was included in several projects, sometimes as distance learning or continuing education for professionals. All projects except two involved PhD students, with a total of 72 for the programme overall. Most projects (*n* = 16) clearly stated that their research group was interdisciplinary and some discussed the benefits and obstacles encountered due to this. A total of 203 articles in scientific journals (including submitted manuscripts) were reported to have been produced during the 6-year period. The widespread target groups and involved organisational levels in the projects’ research activities are illustrated in Table 1. More information on the projects is provided in Additional file 1.

**Table 1:** System/organisational levels where research was performed and levels where support was needed in order to establish the projects and keep them going; the summary provides an overview for comparison

	Project	Levels where research was performed	Levels where support was established	Summary of levels
	2008–2011			
1	ACTION – partnership for increased care and quality	Homebased health and social care Meetings with patients and next-of-kin National networks for IT support	Municipality officers Municipality collaboration	Reg., Org, Unit, Care recipient
2	Bridging the gaps	Micro system (i.e. patient-care provider interaction), Clinical Dep., Diagnosis cohorts, Patient - web support	Micro system, Clinical Dep., Region, National level IT (quality reg.), Municipality IT network (quality reg.)	Nat., Reg., Org, Unit, Care recipient
3	Chronical health	Diagnosis cohort Specialist (MD) cohort	Multiple clinical dep. (several regions) National level IT (quality reg.)	Nat., Unit, Care recipient
4	Innovation systems for better health	Clinical Dep. Hospital or organisation, Region	Clinical Dep. Hospital or organisation, Region	Reg., Org., Unit
5	Sustainability in innovation and organisation learning in healthcare	Patient cohort, Clinical Dep. Hospital or organisation, Region, County	Hospital or organisation Region County	Reg., Org., Unit
6	NDR – Better use of the national diabetes registry (a national quality registry)	Diagnosis cohort, Specialist (Medical Doctor) cohort, Clinical Dep. Hospital or organisation, National level IT	National level information technology (quality reg.)	Nat., Org, Unit
7	Knowledge, management and value creation in geriatric care	Clinical Dep. County	Clinical Dep. County County top management	Reg., Org
8	Increased participation/access to society for people with psychiatric conditions	Meetings with patients and next-of-kin, Diagnosis cohort, Region, National	Municipality Region, National, Non-governmental organisation	Reg., Unit, Care recipient
9	QIHREA - Quality improvement in healthcare, a research and education agenda	Patient, Clinical Dep., Hospital, Region, Region based management network	Micro system Clinical Dep. Hospital, Region	Internat., Nat., Reg., Org.
	2009–2013			
10	Bridging the gaps 2 – Patients as active co-creators in care processes	Micro system National level IT (quality reg.)	Micro system National level IT (quality reg.) Region-based management network	Reg., Org, Unit, Care recipient
11	Care chain – From emergency care to home	Meetings with patients and next-of-kin, Clinical Dep., Hospital management, Municipality management	Clinical Dep. Hospital (management) Municipality (management), Region	Reg., Unit
12	Learning on patient safety	Clinical Dep.	Clinical Dep.	Nat., Reg., Unit
13	FLIP – Atrial fibrillation in primary care	Diagnosis cohort Specialist (MD) cohort	–	Reg., Unit,
14	Nat. guidelines for health promotion – from evidence to clinical practice	Clinical Dep. Professional cohorts in care County, National, Government body	Clinical Dep., Professional cohorts in care, County, National Government body	Nat., Reg., Unit
15	Lean and agile	Hospital County	National research cohort	Org., Unit
16	INTEGRAL	Hospital management	Hospital management University management	Reg., Unit
17	P-Inn – The patient’s innovation system	Patient cohorts National level IT (quality reg.)	National level IT (quality reg.)	Nat., Unit, Care recipient
18	Patient choice system in primary care	Regional	Regional National	Nat., Reg.
19	InOut	Clinical Dep., Hospital National, International	Clinical Dep., Hospital, National level IT, National patient organisation, European Stroke Organisation	Internat., Nat., Reg., Unit,
20	FELLOW – Fellowship program	Clinical Dep.	Clinical Dep. University management	Reg., Unit

*dep.* Department, *Internat.* international organisation, *Nat.* national organisation, *Reg.* regional organisation, *Org.* local organisation, *Unit* clinical department or other organisational unit, *Care recipient* individual patient (incl. next-of-kin)

### Descriptions of collaborative procedures

**Fig. 2:**
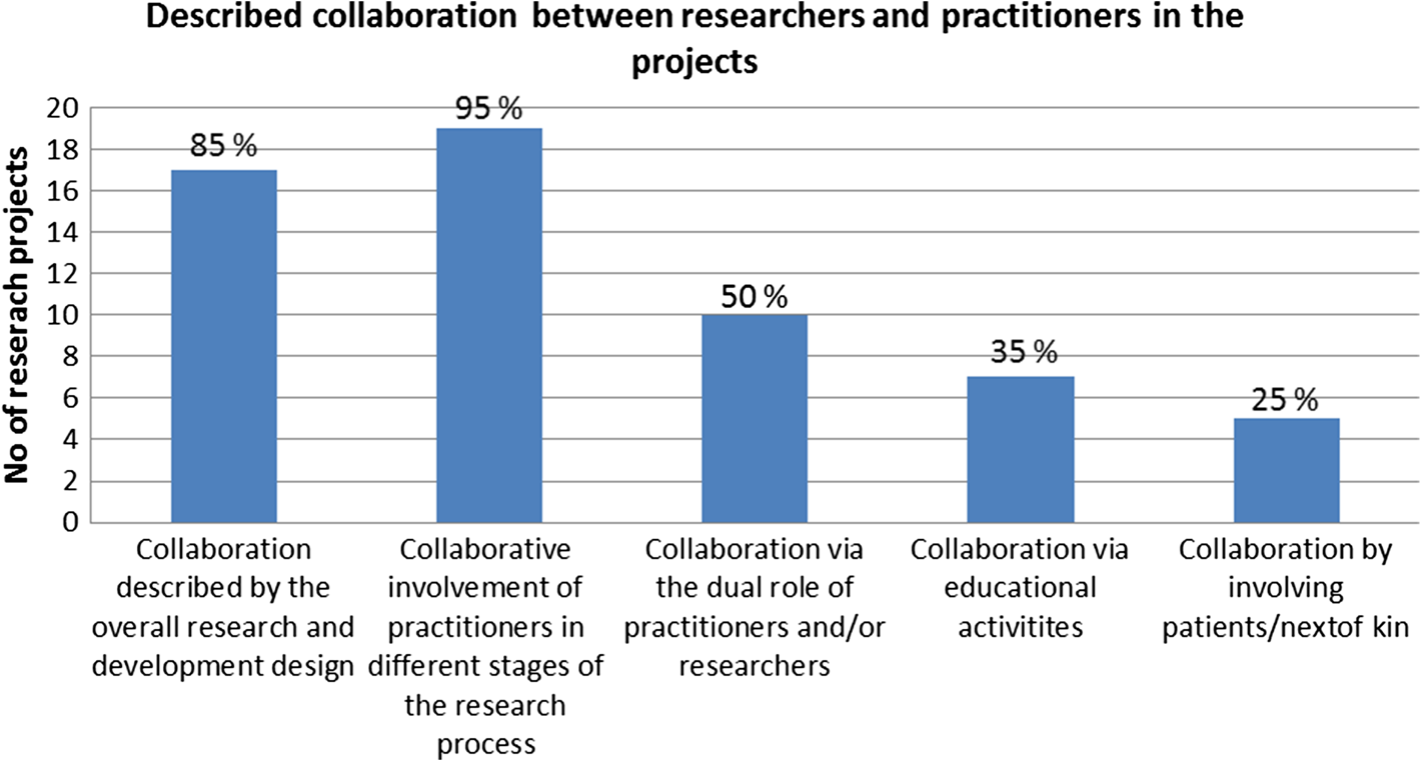
Number and proportion of projects (*n* = 20) providing descriptions in the different subcategories of collaboration

Descriptions of the research collaboration varied – from describing a more or less interactive research design to more explicit descriptions of when and how practitioners were involved during the research process. Practitioner’s and researcher’s boundary-spanning roles served as important bridges between the two contexts. Five main categories were identified to cover the various ways collaborations were described. In Fig. 2, the number and proportion of the projects providing descriptions in each of these categories is provided.

#### Collaboration as described by the overall research and development design

Seventeen projects (85%) clearly described an interactive research design that either involved healthcare practitioners, managers, politicians, patients, next-of-kin or a mixture of those. Seven projects used the term ‘action research’ or ‘action-oriented research’, two used the term ‘interactive research model’, and one used the term ‘participatory design’, all of which imply a close interaction between researchers and practitioners. Examples of statements are “*the project’s action research approach has involved a continuous and iterative collaboration that has both fed into and questioned on-going* [change and learning] *processes*” and “*we used a participatory design where the young people actively participated in the creation of what to study, that is the effects of using a web-based instrument*”. Four projects developed information and communication technology-based solutions in close collaboration with practitioners. The terms ‘co-design’ or ‘co-creator’ were often used in two projects involving patients and their next-of-kin and in a project focusing on mixed learning networks with researchers, patients and other actors.

#### Collaborative involvement of practitioners in different stages of the research process

Interactive forms were described as being used during the following stages: (1) mapping of the research problem and its manifestation in practice, and formulation of research questions; (2) planning, creation of interventions and choices of design; (3) investigation, test, follow-up and implementation processes; (4) analyses, reflection and learning; and (5) reporting on and dissemination of results, including further implementation of studied intervention. Nineteen projects (95%) described an interactive approach in at least one of these stages. Collaboration or interaction was most common during follow-ups and feedback processes (Stage 3 and 5), and less interaction was described during the formulation of the research problem and during analyses (Stage 1 and 4). Statements like “*conducting research with practitioners, not on practitioners*” and “*patients have participated in the mapping* [of the current situation]” are examples of Stage 1. Stage 2 is exemplified by the statements “*practitioners participated during the construction of the interview* [manual]”. Statements such as “*the researchers and contact persons* [from the healthcare organisations] *have met 1–2 times a year to discuss and reconcile research questions, data collections and results from different sub-projects*” represent several stages (Stage 1, 2, 3 and 5). Involving practitioners directly in analyses (Stage 4) was not explicitly mentioned, but the described interactive sessions could involve the discussion and validation of results. Statements such as “*the researchers regularly fed back their observations and analyses to the hospital management and hospital unit representatives*”; “*we had several formal and informal feedback sessions and this feedback has been further used*”; or “*the feedback and the action research approach have given researchers an opportunity to stimulate reflection and contribute with knowledge on implementation, learning and change*” can represent situations when practitioners participated in the interpretation of results and that this had effects on both research and practice. Thus, if researchers in the research teams holding dual roles are not accounted for, no project involved non-researchers in the entire research process.

#### Collaboration enhanced by the dual role of practitioners and/or researchers

Ten projects (50%) described a dual, boundary-spanning role held by either the practitioners or the researchers. This dual role of being involved in both practical work and research was more common for clinicians performing research in the area of their expertise, e.g. in projects and sub-cases with a clinical focus. This can be exemplified by statements such as *“we are conducting research with actively serving practitioners and clinicians*” or as a statement made by a clinician project leader “*improvement work for increased quality in healthcare is best performed directly in connection to the meeting between patients and professional care-givers*”. In one case, in the Clinical Innovation Fellowship programme, teams of practitioners were trained as action researchers for 2 months (fellows) and spent 6 months working in clinical quality improvement in healthcare, sometimes in collaboration with students working on their masters or bachelor thesis. Examples on practitioners’ dual role were described as “*some healthcare staff became PhD students and thereby gained a double bridge-building role*” (e.g. conducting research within their organisation). The role of translating knowledge as part of the dual role was exemplified as “*we have involved healthcare staff as interpreters*”. No further details on what this actually meant were provided. Three projects described that their research team consisted of both researchers and practitioners.

#### Collaboration via education

Seven projects (35%) described interactive processes linked to educational activities, either designed for enhancing learning and (sustained) interaction between researchers, practitioners and their organisations, or designed for students to participate in and study development initiatives while learning together with practitioners. Some involved the development of new programmes and courses where “*experience-based education*” was essential or where networks of previous healthcare students (now as practitioners) were established as a resource. Some examples concerned the interaction between students and practitioners exemplified by statements such as “*collaboration with students concerning knowledge on improvement, using a model for learning that involves multi-professional teams with care professionals and students that together reflect on the potential improvements of the care practices*”, or the involvement of students in the development of healthcare practices exemplified by one project’s new master’s programme where conducting an improvement project in practice was a basic requirement for a master’s thesis.

#### Collaboration by involving patients/next-of-kin

Five projects (25%) described an active involvement of patients in the research process. One project developed mixed learning networks with both patients and their next-of-kin in an active learning process. Another project had “*engaged patients, relatives and care professionals in the work of changing care practices*” describing patients as “*co-creators, co-producers and co-evaluators*”. Two projects had developed information and communication technology solutions together with patients and one project had given patients the opportunity to conduct single randomised controlled trial (RCT) studies using their own measurements.

### Challenges, obstacles encountered and the main lessons learned from the research collaborations

**Fig. 3:**
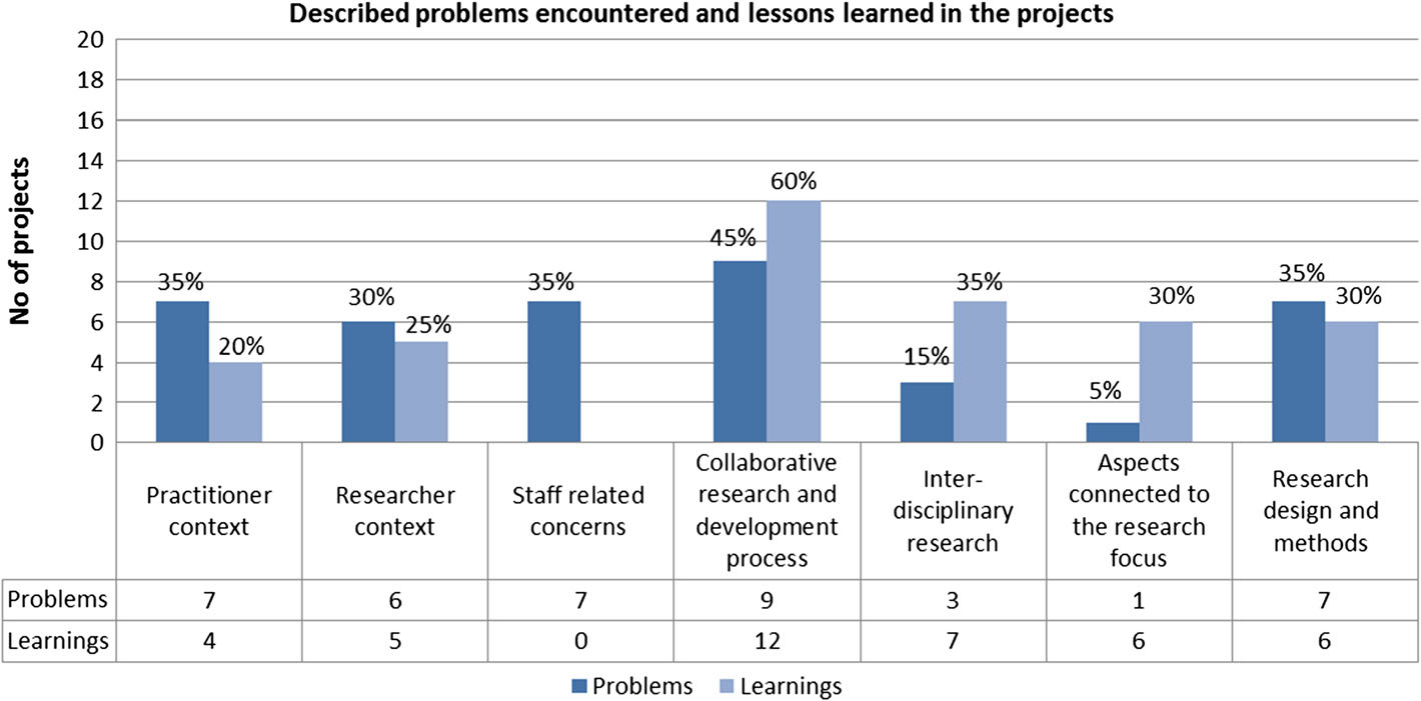
Number and proportion of projects (*n* = 20) providing descriptions in the subcategories of problems encountered and lessons learned

Seventeen projects (85%) provided detailed descriptions of problems encountered and a meta-reflection of lessons learned. The others reported no problems or none that could not be dealt with and/or interpreted the question on lessons learned as an opportunity to report on detailed project results. We could not find any patterns for the two projects reporting no problems (19 and 20). Described problems were classified into six categories and similar categories also summarise the lessons learnt, except for ‘staff-related issues’, which was mentioned only as a problem. In Fig. 3, the number and proportion of the projects providing descriptions in these categories is presented. The most reported problem category concerned the collaborative and partnership research and development process (with practitioners) followed by issues related to the practitioners’ context and the research design and methods used. Thus, most problems described were related to the adaptation to or collaboration with practitioners and their organisations and the different agendas of and demands from the practitioners’ and researchers’ context. It was sometimes difficult to maximise the fit between the project’s agenda, the research process and the dynamic agendas and activities of the organisations and their representatives. A third of the projects reported on problems within a single context or issues related to research staff or designs. Most of the lessons learned also concerned the collaborative and partnership research process. Otherwise, lessons learned were more evenly spread, except for the staff category.

Reported problems related to ‘the practitioner context’ concerned partner engagement, competing activities, economy, political decisions and organisational changes. Examples of problems were “*delays, organisational changes and changes in management teams that altered the initial engagement in the project*”; “*other parallel development projects have taken time for our collaborating organisations*” and “*the development unit that was to coordinate interventions within the county council was closed down*”. The major lessons learned mirrored these problems, acknowledging the anchoring, flexibility and time needed to work with the practitioners’ (politically governed) organisations. Examples of statements provided are “*county councils and universities are large organisations where change takes time and anchoring on several levels must be secured*” or “*changes in health and social care occur quickly and are difficult to foresee and improvements have to adjust to this, and to document such periods is both a challenge and an opportunity*”.

Reported problems related to ‘the researcher context’ often concerned the PhD, master or bachelor level students involved, where university demands caused delays, frustration or extra work. Examples of statements were “*writing applications to fully finance participating PhD students have taken a lot of time and energy*”, “*the full student participation was delayed and affected the project*”, and “*the choice to build a school for research studies have delayed publications*”. Learnings included the need to deal with such aspects and the demands this put on the project management and the research group, exemplified by “*the research field is young and it has been central to build networks and cooperation with others*” and “*to build research and education demands active and competent project management, not only an effective administrative management but also an overarching research perspective, and this administration and research overview is time demanding*”.

The category ‘staff-related concerns’ contained human resource management issues related to research group members (mainly researchers and PhD students) and no lessons learned related to these were recorded. Issues concerned interruptions or delays in the project process due to recruitment of staff, leave due to sickness, parental leave or other changes in work situation. Example of statements were, “*one researcher was on leave for a long-time due to sickness and this made us have to start all over again with analyses*”, “*the project leader was assigned a mission by the government*”, and “*two of the project’s PhD students went on parental leave for a total period of 3 years, which delayed the project*”.

Problems related to ‘the collaborative and partnership research and development process’ concerned the time and energy needed for the process, expectations on the practitioner organisation, and differences in focus and pace of the more rigorous scientific process compared to the quicker decision-processes in the practitioners’ contexts. Statements such as “*the interactive research process takes more time to establish*”, “*output data were supposed to be generated and provided by the county council, we had to use whatever we could find when this could not be done*”, and “*our organisational partners’ need for quick results and tendency to change work approaches towards the introduced intervention made it hard to scientifically evaluate the results*”. The lessons learned described were related to insights concerning different views, contexts and efforts to handle dilemmas. Example of statements were, “*research and practice are two systems with a different pace, demands on PhD students delay analyses and cause delays in the feedback to practitioners*”, “*practitioners sometimes have un-realistic expectations on researchers*”, and “*the importance as a researcher to always be prepared to re-evaluate, be flexible and adapt the project focus and time plan after the dynamic situation of the empirical reality studied*”. Some important actors in the researcher context made this process difficult, exemplified by “*the ethical board approving the project has demanded a clear distinction between research and improvement work*”.

Problems related to conducting ‘inter-disciplinary research’ gave examples of paradigmatic differences in perspectives, assumptions, experiences, methods and ways to report results. Examples of statements were “*PhD students struggled against time and felt fragmented and torn between their own research area and the demand for interdisciplinary research*” and “*to form such group consisting of different disciplines takes a long time and the mixed methods approach demanded a period of interactive learning for the interdisciplinary research group*”. Lessons learned related to conducting interdisciplinary research concerned insights into the process of building interdisciplinary teams and benefits of several perspectives, for example, “*interdisciplinary research is not easily or quickly established, it takes a long time for participants to build trust and understanding of each other’s perspectives and terminology, and to be attentive and responsive towards each other’s contributions*” and “*the importance of a firmly grounded theoretical framework and the creation of instructions for the research activities during an interactive process*”.

Problems connected to ‘the research focus’ were reported by one project and concerned the investigated health economic concept, which was new to the area and demanded a shift in perspective. Six projects reflected on lessons learned connected to the research focus, for example, “*internet-based support and coaching have provided coaches with increased insights into how large a disability individuals with neuropsychiatric disabilities can actually have*” and “*the care structure is a central factor for improvement and the availability of stroke units essential*”.

Problems related to the chosen ‘research design and methods’ concerned demands of specific methods and instruments, for example, “*building a computer model and the inclusion in the RCT study has taken more time than anticipated*”, “*the ethno-methodological observation method is time and resource consuming*”, and “*it was challenging to find data for follow-up studies and a lot of work studying patient journals*”. The reported lessons learned concerned the chosen approach, design or spread of interventions exemplified by the following statements: “*the meta-study has been important for synthesising and extracting knowledge*”, “*action research is an important method for gaining new knowledge and for stimulating participation in improvement initiatives*”, and “*the major lesson learned has been that the work to implement findings does not start or continue by itself, it will demand large efforts and continuing economic resources*”.

## Discussion

### Addressing complex research areas in complex systems affects the complexity of the collaboration

The research programme was set out to address problems related to the gap between research and practice in health and social services. Several projects reported putting large efforts on addressing this broad and interdisciplinary content by trying to frame clinical and service activities within general organisational frameworks. Many projects focused on how to organise care to achieve a more research-based practice or to identify hindrances, contradictions or opportunities related to development. To study such complex phenomena over several system levels often requires longitudinal designs, interdisciplinary approaches and a mix of methods [55], in combination with participatory approaches (e.g. [15]). A large variation of research and methodological approaches, most of them very demanding, was used in the projects, from action research to quasi-experimental studies and RCT designs. Single or multiple case studies using qualitative and mixed methods were common. The use of a demanding research design and the lack of a research culture on behalf of practitioners and their organisation can act as a strong barrier for research collaboration [56].

Research use represents a specific form or knowledge utilisation [57, 58], where research findings support decisions through a complex process enacted at a practical level. Scott-Findlay and Golden-Biddle [59] argue that understanding this process only at an individual level is misguiding and should be complemented with an understanding of practitioners’ research use at an organisational level. When making major changes in line with new research knowledge the authors propose strategies involving efforts to change organisational culture and consideration of the organisation’s values and assumptions. This indicates the need for researchers to have an in-depth understanding not only of the involved practitioners as individuals or professional groups but also of their organisations in order to enhance the use of research findings.

A majority of the projects addressed several levels of the involved healthcare organisations (or systems) implying several types of partners and stakeholders to interact with. Demands put on project management and the project group corresponded with these multidimensional views and mixed approaches – providing variable room and energy to support and adhere to demands related to collaboration and research use. The importance to understand and address the interaction and inter-connection between system levels during development efforts has been highlighted by several researchers [60, 61]. The types of project management, research design and collaboration across the organisational system that are needed to get access, and to build, co-create and transfer knowledge in order to enhance development in organisations have been less discussed. Recently, the heterogeneity among knowledge users and the need for relationship brokering in collaborative and partnership research has been highlighted [38].

### Different perceptions of interdisciplinary, collaborative and partnership research and on the roles and relationships of involved actors

Any empirical research process is characterised by interaction between the researcher and practitioner context, each one in a constant flux, continuously changing and restructuring. The research process in itself also differs depending on the type of research and focus, for example, if there is an innovative or developmental component involved to be tested and evaluated or if an on-going situation or phenomenon is investigated. Qualified practitioners, sometimes enrolled as PhD students, served as knowledge brokers interpreting results – in both directions – when understanding the practical phenomenon in theoretical terms and when translating theories and models used into practical terms. Educational fora and interactive learning approaches were a significant part of many projects, often combined with active involvement of practitioners. Involvement of patients and their next-of-kin, often during intervention design, was also described.

Based on our findings, using the terms introduced by Sibbald et al. [49], two projects could be described as having a more researcher-dominant or token partnership, while eight projects involved non-researchers to some extent in an asymmetric partnership and ten projects had features that indicated a more egalitarian partnership.

**Fig. 4:**
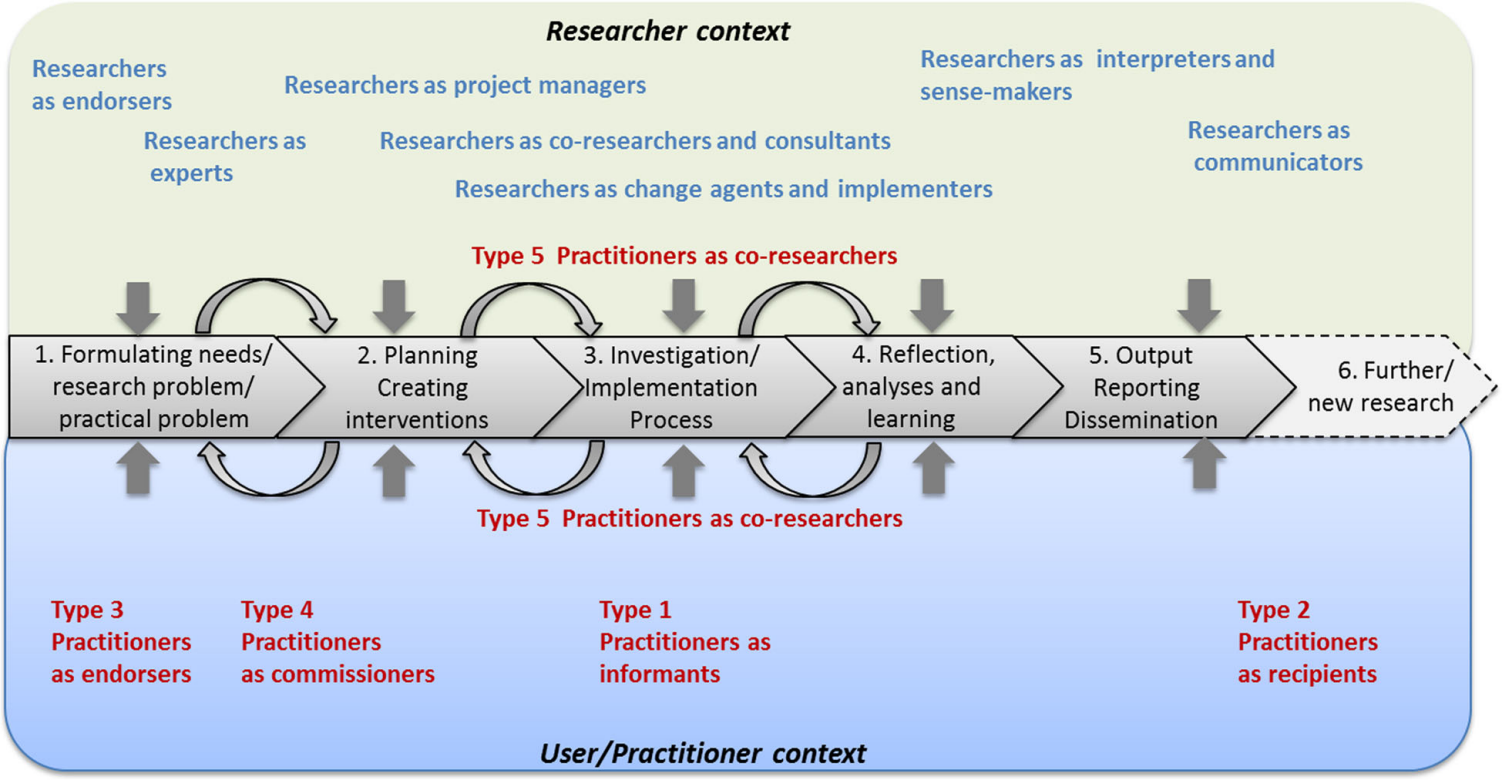
The five types of potential roles of practitioners during a research process according to Martin [44], and some suggested potential roles for researchers to enact

The role of the researcher varied – from being deeply and practically involved in a development process in the practitioner context, to more distant when studying the effects of different care choice models. Martin [44] listed the various roles of practitioners in research collaborations, but researchers are also able to enact different roles, depending on opportunities, preferences and the chosen research focus (explorative, descriptive, explanatory, intervention and action oriented). An ability to describe the nature of both the practitioners’ and researchers’ roles may indicate the type of interaction, participation, involvement or influence that can be expected from both parties. In Fig. 4, the potential roles of practitioners [44] are displayed together with some suggested potential roles for researchers to enact. Indications of egalitarian partnership were identified in projects that clearly described interactive research approaches, but otherwise our data did not provide much detail on the relationships and roles. For an in-depth study of the enactment of different roles over time, situation and context would provide more information on how the researcher–practitioner relations evolve over time, both initially and in long-term partnerships.

Interdisciplinary research, often across faculties, was enhanced, further developed and deepened in the majority of the projects. There were several examples of clinical researchers working together with both social scientists and scientists from technical faculties. Building trust and lessons learned concerning different perspectives and ways of conducting research were important. The programme’s goals regarding the expected development of institutional learning structures and establishment of research on how to lead, manage and develop practices in health services organisations might have aided this development, more so than traditional programme calls.

Creating long-term relationships between researchers and decision-makers might be a useful way for bridging research and practice. For such a strategy to become successful it has been argued that it must be complemented with strategies for involving researchers in decision-making around policy and practice and with core funding for building and upholding capacity for knowledge exchange [24]. Maintaining such relationships for longer periods often requires formal support or structure. The national collaborative examples (e.g. practice-based research networks) described in the introduction can be one way to achieve this. Incentives for supporting and nourishing such relationships are scarce in the research context where funding is often difficult to obtain and outcomes are measured in production of scientific publications. Supporting the costs associated with research involvement (for both parts) can facilitate partnership with managers and decision-makers [34]. This is an important message to research funders, who despite tasks to increase “*research utilisation and interaction with society*”, often support short-term projects with more limited scopes – a strategy that is seen as insufficient for sustaining practitioner relationships and achieving goals of research utilisation [24].

Research collaboration between researchers and decision-makers and other types of stakeholders can be ethically challenging, especially when members of the team are insiders or participants in a studied case. The role of insiders needs to be clarified, for example, with regards to access to raw data and ensuring anonymity and confidentiality [62], but also a sensitivity on how to handle organisational information as a researcher. Hofmeyer et al. [62] recommend the use of self-reflection and ethical dialogues to enhance shared meanings and understandings among researchers and decision-makers – a practice that requires time, motivation and skills, and which could be highlighted in future programmes aiming for collaborative and partnership research.

### Experienced challenges, obstacles and enablers for interdisciplinary collaborative, partnership research

There were indications of the tension between the demands from the practitioner versus the researcher contexts and the role of the researcher. Dilemmas occurred, for example, when adhering to conflicting demands for knowledge translation and the different types of knowledge production [63], such as a curiosity-driven inquiry based on a positivist epistemology or a problem-solving epistemology with knowledge production in the context of application [64].

Collaboration in the projects’ research constellations was mentioned both as an asset and a challenge, especially the interdisciplinary aspect where team members might differ in views and ways to handle collaboration. That interdisciplinary research is challenging is not a new insight (e.g. [65, 66]) and there are strategies to facilitate such research, i.e. selective collaboration, cross-training, sustained relationships, good humour, participation in peer review, declaring the place of one’s work, and balancing dissemination of research between peer and other audiences [67]. Past experience of interdisciplinary collaborations and an understanding of different views on epistemology are foundations that can enhance collaborations. There were indications in some projects that participating researchers did gain a deepened appreciation of the need to join several paradigms in order to understand the complexities of the issues at hand. The ‘inside’ clinical PhD students could act as door openers and knowledge brokers [68] to the world and practice of health and social services for the social or technical sciences PhD students, and vice versa. Clinical researchers and PhD students were invited to new knowledge paradigms by social scientists. Other practitioners involved as co-researchers in the projects could employ various brokering strategies, e.g. to engage, collaborate and build capacity [43]. The described challenges for these persons to adhere to expectations and demands from several contexts in their in-between role and the conflicts that follow when demands are incompatible or role expectations are ambiguous have also been identified elsewhere (e.g. [40, 68]). Previous research has shown that innovative researchers tend to be more engaged in research collaborations, both disciplinary and interdisciplinary, than adaptive researchers who prefer to work with well-established procedures within existing frameworks and in stable groups [69, 70]. Female scientists are also more engaged in interdisciplinary research, while years of research experience has been found to be positively correlated with collaboration both within one’s own discipline and with researchers from other scientific disciplines [70, 71].

Some of the problems and enablers experienced in the projects correspond to the enabling factors in the research partnership process as described by Sibbald et al. [49] and Rycroft-Malone et al. [36]. The described difficulties due to changes occurring in the partner organisation, role problems due to ambiguous or conflicting demands, and different view and paces for knowledge production and use between researcher and partner organisations correspond to the challenges described by Sibbald et al. [49] (Fig. 1). Problems and lessons learned related to time needed for development of mutual trust and respect, power issues, and planning and implementing change are in line with Rycroft-Malone et al.’s [36] observations (Fig. 1). Skilled project leaders and researchers able to handle various perspectives and enhance communication among involved actors and the establishment of infrastructures and long-term relationships, as in the sustainable collaborative structures initiated or strengthened by the projects, are all considered as enablers for research partnership [49].

Ideally, there is room for a mixture of research approaches in a programme, in line with current debates on mixed methods where Gorard et al. advocate the development of a research community where “*all methods have a role, and a key place in the full research cycle from the generation of ideas to the rigorous testing of theories for amelioration*” ([72] p. 162, [73]). Collaborative approaches are amendable to many different research topics, designs and disciplines, and the mixture of projects, research approaches and collaborations in the programme provides an example of this. It also shows the need to consider how to evaluate research applications when broader, complex issues are the topics, as also highlighted by others [38]. Assessment of the benefits and lessons learned through participating in the research process for individuals and partnership organisations, as well as of the sustainability of partnerships and joint interventions, might be a way forward.

### Limitations of the study

The study mainly represents project leaders’ and senior scientists’ views on the research programme and their own project’s efforts to bridge research and practice and initiate collaborative and partnership research. No representatives of the involved practitioners were interviewed and the reporting on impact and collaboration with stakeholders was retrieved via project documents and interviews with representatives of the projects. To expand the understanding of the entire process and all actors’ perspective on the acquired knowledge, the partners’ views will need to be addressed. We also acknowledge that there might be information missing in final reports depending on the amount of text provided and how questions in the report template were interpreted, as well as the potential bias introduced by providing ‘socially accepted’ information to highlight the project’s benefits. We sought to reduce this bias by asking representatives from all projects to read through the results of analyses and comment on any missing information or misunderstandings – all projects provided answers and five of them provided additional information or corrections. Frequencies and proportions were calculated to indicate trends and overall patterns and should otherwise be interpreted with caution. Due to resources, it was no possible, but it would be interesting for future research, to address the actual impact of the collaborative and partnership projects on healthcare practices, on further collaboration, and in terms of cultural change, research use and relevance of the research conducted [49], despite the potential challenges posed by the projects’ diversity.

## Conclusions

Using collaborative efforts to perform research on complex areas in complex systems requires a contextual understanding, longitudinal efforts, collaboration on multiple system levels and often interdisciplinary designs. Described problems, mirrored in the lessons learned, provided an indication on challenges to manage interdisciplinary, collaborative and partnership research, enact different roles and bridge several worlds as a researcher. Staffing, funding PhD students and paying attention to the work environment are some of the duties adhering to the tasks of an employer. Administrating and handling the project budget and monitoring its progress belong to the role of a project leader/administrator. Possessing knowledge on research designs and on the characteristics, demands, etc. of several disciplines is important in interdisciplinary, collaborative and partnership research. Finally, skills in collaboration and communication are needed, including a basic understanding of both the researcher and the practitioner contexts, while not forgetting any ethical concerns. Such demands and the often ambiguous roles and conflicting expectations make the research process challenging.

Funders, as well as managers, practitioners and researchers, might underestimate the complexity induced and efforts needed to collaborate during a research process, especially in multifaceted and complex research areas. Both interdisciplinary research teams and researcher–non-researcher teams can be challenging and time-consuming per se. By mixing these two conditions the situation becomes exponentially complex, as everyone has to learn about each other and adapt in various ways. The development of support from various decision-makers and build trust and understanding with involved practitioners at several levels of a healthcare system/organisation will need both skills in and arenas for communication and interaction. For the researchers, this takes time and energy from actual work with data collection, analyses and scientific writing. For the practitioners, this puts demands on understanding a research process and how it fits with ongoing organisational agendas and activities, and allocating enough time. Nevertheless, ensuring good relations (relationship brokering) is an important precondition for establishing a research process and gaining access to high quality data, especially on complex issues.

Another process that might be overlooked is the formation and building of research teams and the enactment of different roles in research intending to be both interdisciplinary and collaborative. If collaboration is not already established among researchers and involved partners, the experiences show that these processes need significant time and effort on behalf of both researchers and practitioners. Such efforts must not be underestimated if project agendas and schedules are to be realistic. The different roles and skills and the time required by researchers to both conduct research and contribute to the solving of complex problems in society by forming interdisciplinary research collaborations and collaborations with decision-makers and practitioners may be underestimated or simply ignored by involved stakeholders. Many researchers (especially PhD students) are not trained or experienced in working with interdisciplinary research teams or in a collaborative way with practitioners. Practitioners, in turn, may lack experience and skills in research collaboration. Both these aspects may have contributed to the problems encountered in this study. The lessons expressed can thus provide input for future collaborative or partnership research initiatives.

Research funders, as well as researchers and partners, may also benefit from gaining an overall understanding of the different types of research that can aid an understanding of and support changes in health and social services – from explanatory studies and experimental research to explorative studies and case study research, as in this case, which focused on understanding larger systems and more complex phenomena. Moreover, a flexibility regarding content and schedule is necessary to meet the complex demands, particularly concerning the time and resources needed for project management. To avoid exhaustive situations for involved researchers and practitioners, such considerations need to be included in the agenda of the funding body.

There is a need for more empirical studies on the conditions for researchers and practitioners in collaborative partnership and interdisciplinary research processes with the aim to increase the capabilities in addressing complex questions and the ‘usefulness’ of research in practice. An assessment of the efforts made to handle the different contexts and views of all involved actors in interdisciplinary, collaborative and partnership research initiatives in greater detail would provide more information on such processes and on their outcomes. Future studies could also address some remaining questions, including do the efforts to build interdisciplinary, collaborative and partnership research lead to better uptake and use of research outcomes, or provide more useful outcomes for practitioners and patients? Do they lead to deeper learning and understanding for researchers, and does the bilateral learning process and integrated knowledge translation between practice and academia occur?

### Comments on the results of the national programme

The programme’s goals can be considered as new and innovative in the Swedish context. Further, the programme chose to fund less traditional research projects such as intervention studies, studies of natural experiments and the building of new infrastructures. This approach may be risky with regards to results evaluation, but the rich variety of projects, foci, new structures and lessons learned provided more types of results than traditional ones (i.e. scientific presentations and publications), which fits rather well with the initial broad aims of the programme. Scientific production, measured through traditional metrics, was substantial. Other presented results were categorised into five areas of innovation (what to develop) – a product/artefact, an approach when meeting patient/next-of-kin, routines and work procedures, administrative systems and structures, and increased organisational learning/competence. All projects reported results in at least two categories, and six projects reported results in all categories. Some of these were tangible, like employment of nurses in new roles, education of hundreds of care providers, new IT systems, web portals, academic courses, and a decision support used yearly in 25,000 patient meetings; some results were also very vulnerable. Quality in care processes can take long time to develop, especially when many actors and interests are involved, but might be destroyed by one major politically decided organisational change.

## Additional file

Additional file 1: Overview of the projects based on information in documents. (DOCX 19 kb)
