# Supplementary material for: Collaborative and partnership research for improvement of health and social services: researcher’s experiences from 20 projects
Source: Health Res Policy Syst. 2018 May 30;16:46. doi: 10.1186/s12961-018-0322-0 (PMC5975592; doi:10.1186/s12961-018-0322-0)
Supplement: Supplementary file 1 — Overview of the projects based on information in documents. (DOCX 19 kb) [file 12961_2018_322_MOESM1_ESM.docx]

Additional material 1: Overview of the projects based on documents (the interviews provided additional information on research design and methodology) i.e. project name and description, funding, research methodology, no of PhD student, and interdisciplinary research teams (i.e. researchers from more than one scientific faculty).

|  | Project | Description | Methodology (as described) | No of PhD students | Inter-disciplinary research team |
| --- | --- | --- | --- | --- | --- |
| 1 | ACTION – working in partnership for increased care and quality (2008) 5 MSEK | Increase quality of care/service for old people with chronical conditions living at home and their care takers by the use of Information and communication technology (ICT) and Mixed learning networks | Multiple methods | 0 | Not clearly described |
| 2 | Bridging the gaps (2008)  9 MSEK | Identify, investigate and develop models over critical gaps that hinder provision of best possible care. Focus: learning and information logistics. Develop a regional research and educational platform close to clinical practice. | Interactive research model, Case studies (8) Multiple methods | 12 | Yes |
| 3 | Chronical health (2008)  4 MSEK | Investigates how knowledge bearing internet services can be implemented, integrated and used by patients and care providers to improve care. Modelling and simulation as tools and test of two care models. | Two sub studies  One RTC study, Multiple methods | 1 | Yes |
| 4 | Innovation systems for better health (2008)  11 MSEK | Investigation of 12 local learning structures focusing on implementation, use and relations between innovation, implementation, and consequences | Action research Case studies (12), Multiple methods | 8 | Yes |
| 5 | Towards sustainability in innovation and org. learning in healthcare (2008) 4 MSEK | Investigate two attempts to build sustainable organizational learning structures in complex regional health and social care systems – a learning organization structure and a regional health promotion program | Action research Case studies (2cases/12 sub stud.) Multiple methods | 2 | Yes |
| 6 | NDR -Better use of the national diabetes registry (2008)  3 MSEK | Develop ways to increase the use of the national quality registry for diabetes for improving the quality of diabetes care. | Three sub studies | 3 | Not clearly described |
| 7 | Knowledge, management and value creation in geriatric care (2008)3 MSEK | Investigate how knowledge is translated to local context and situations by investigating how and when different types of knowledge are translated to practical care situations. | Two sub-studies | 0 | Not clearly described |
| 8 | Increased participation and access to society for people with long-term psychiatric conditions (2008) 2 MSEK | Evaluate the process and effects on young patients with psychiatric disorders and their relatives of a co-created IT-solution’s in the form of a web-support providing information and education | Three sub-studies, participative design, Multiple methods | 2 | Not clearly described |
| 9 | QIHREA Quality improvement in healthcare - a research & education agenda (2008) 9 MSEK | Establish an infrastructure/a knowledge platform with action oriented education and research on improvement, transformation and knowledge mediation. | Action oriented research Applied sub projects (3 projects + some smaller) | 10 | Yes |
| 10 | Bridging the gaps 2 - The patient as an active co-creator in care processes (2009) 4 MSEK | Demonstrate methods and tools to achieve better results in daily clinical practice with a focus on the patient as a co-creator, co-producer and co-evaluator of care and care services. Create a platform for learning. | Interactive research model, 13 sub projects Multiple methods | 9 | Yes |
| 11 | Care chain - From emergency care to home - (2009) 5 MSEK | Implementation and evaluation of an intervention for old people with multiple health conditions. Develop and implement a care program for all care levels – hospital, primary care, municipality home healthcare/service. | RCT study | 5 | Not clearly described |
| 12 | Platform for learning on patient safety: a basis for a positive spiral (2009)  4 MSEK | Contribute to better patient safety by developing, evaluating and studying a system that improve learning and safety in health care. Create a structure/platform in which sub projects would lead to double loop learning on methods used in participating units. | Three themes, 11 sub projects | 3 | Not clearly described |
| 13 | FLIP – Atrial fibrillation in primary care – New methods for stroke prevention (2009)5 MSEK | Test of models for screening of patients in order to prevent stroke by identifying atrial fibrillation. | Three sub projects, one RCT with control group | 2 | Not clearly described |
| 14 | National guidelines for health promotion – from evidence to clinical practice (2009)  6 MSEK | Investigate the processes of identifying evidence-based knowledge, the process of summarize, prioritize, and implement such knowledge in health care. Investigate the challenges of developing a more health promoting health car in Sweden. | Four sub-studies, Case studies (6) , Multiple methods, | 3 | Yes |
| 15 | Lean and Agile (2009)  5 MSEK | Investigate under which circumstances a health care org. can use logistics and experiences from other sectors and both be flexible and adjust to changes (agile) and improve the use of resources, results and quality (lean). | Case study, Multiple methods, Action research | 3 | Yes |
| 16 | INTEGRAL (2009)  4 MSEK | Organizational support for integration of research and practice based knowledge or innovation processes, generation of individual and organizational learning both with a long-term objective of achieving more effective and purposeful healthcare. Student participation in improvement projects, Patient safety dialogue, Method councils were evaluated. | Three subprojects and one meta study | 2 | Yes |
| 17 | P-Inn – The patient’s innovation system – for better health by the use of quality secured knowledge (2009)5 MSEK | Development (co-production with patients) and evaluation of services for patients that creates and passes on quality secured knowledge that aim for better health for the individual. Study of the open innovation processes required if patients shall be able to participate in the creation of new services. | Four sub projects, Action research, Co-design, Multiple methods | 1 | Yes |
| 18 | Patient choice system in primary care (2009)5 MSEK | Evaluation of different care choice model in primary care. Investigation of implementation obstacles and effects of care choice model in primary care. | Six sub studies. Multiple methods | 1 | Yes |
| 19 | InOut (2009)  3 MSEK | To implement new and sign out old methods concerning stroke. What works and how is healthcare quality affected? The project follows how important quality indicators develops over time in different areas of stroke care | Case studies, Multiple methods | 3 | Yes |
| 20 | FELLOW - KTH-KI-SLL Fellowship program (2009)  4 MSEK | Creation of a sustainable structure for knowledge transfer and development of clinical practices. Evaluate if fellowship is a feasible method for affecting the innovation climate in a hospital. Teams of fellows and also students’ thesis work connected to clinics to aid improvement | Not clearly described | 1 | Yes |
